# A safe-enhanced fully closed-loop artificial pancreas controller based on deep reinforcement learning

**DOI:** 10.1371/journal.pone.0317662

**Published:** 2025-01-27

**Authors:** Yan Feng Zhao, Jun Kit Chaw, Mei Choo Ang, Yiqi Tew, Xiao Yang Shi, Lin Liu, Xiang Cheng

**Affiliations:** 1 Institute of Visual Informatics, The National University of Malaysia (UKM), Bangi, Malaysia; 2 Faculty of Computing and Information Technology, Tunku Abdul Rahman University of Management and Technology, Kuala Lumpur, Malaysia; 3 Department of Endocrinology, Zhengzhou University People’s Hospital, Zheng Zhou, China; 4 College of Information Engineering, Henan Vocational University of Science and Technology, Zhou Kou, China; King Fahd University of Petroleum & Minerals, SAUDI ARABIA

## Abstract

Patients with type 1 diabetes and their physicians have long desired a fully closed-loop artificial pancreas (AP) system that can alleviate the burden of blood glucose regulation. Although deep reinforcement learning (DRL) methods theoretically enable adaptive insulin dosing control, they face numerous challenges, including safety and training efficiency, which have hindered their clinical application. This paper proposes a safe and efficient adaptive insulin delivery controller based on DRL. It employed ten tricks to enhance the proximal policy optimization (PPO) algorithm, improving training efficiency. Additionally, a dual safety mechanism of ’proactive guidance + reactive correction’ was introduced to reduce the risks of hyperglycemia and hypoglycemia and to prevent emergencies. Performance evaluations in the Simglucose simulator demonstrate that the proposed controller achieved an 87.45% time in range (TIR) median, superior to baseline methods, with a lower incidence of hypoglycemia, notably eliminating severe hypoglycemia and treatment failures. These encouraging results indicate that the DRL-based fully closed-loop AP controller has taken an essential step toward clinical implementation.

## Introduction

Individuals diagnosed with type 1 diabetes mellitus (T1DM) face challenges due to inadequate or absent insulin secretion, necessitating vigilant monitoring of blood glucose levels (BGL). Extracorporeal insulin injections are employed to maintain stable BGL [1]. However, excessive insulin administration can induce hypoglycemia, characterized by dangerously low BGL, leading to immediate symptoms such as drowsiness, tremors, confusion, loss of consciousness, seizures, and in severe cases, coma or fatality [2]. Conversely, insufficient insulin levels result in hyperglycemia, evidenced by elevated BGL, contributing to the onset of chronic complications such as retinopathy, nephropathy, and neuropathy [3]. Consequently, devising personalized insulin management strategies tailored to individual drug responses has emerged as a focal point in recent research endeavors within related disciplines.

The artificial pancreas (AP) is widely recognized as one of the most promising advancements in diabetes treatment and management. Its objective is to provide an alternative to manual insulin injections for individuals with diabetes by simulating and augmenting the pancreas’ natural hormone production. An AP system typically consists of three key components: a continuous glucose monitor (CGM), an insulin pump, and a controller that emulates the glucose regulation function of a healthy pancreas. Recent advancements in continuous glucose monitoring (CGM) and insulin pump technology have shifted the research focus toward developing closed-loop control algorithms for artificial pancreas (AP) systems. The goal is to enhance the autonomous regulation of blood glucose levels (BGL) by creating a closed-loop connection between insulin pumps and CGM sensors to maintain the patient’s glucose concentration within the normal range of 70–180 mg/dL or 3.9–10 mmol/L [4], ultimately improving the quality of life for individuals with type 1 diabetes mellitus (T1DM) [5]. Various nonlinear control strategies have been successfully implemented as control algorithms in AP systems for managing BG in T1DM patients. These include model-based approaches such as Model Predictive Control (MPC), Adaptive Control, Sliding Mode Control, Backstepping Control, Feedback Linearization-based Control, and Synergetic Control, data-driven methods like Fuzzy Logic Control (FLC) and neural network-based approaches, as well as hybrid control strategies integrating multiple ways [6]. Coupled with CGM and insulin pumps, these algorithms can address the challenges of nonlinear glucose regulation under uncertainty. Highly reliable algorithms such as the MPC and the linear control algorithm Proportional Integral Derivative (PID) have been incorporated into commercially available AP systems approved by the U.S. Food and Drug Administration (FDA).

Beyond the challenges of uncertainty and nonlinearity, the complexity of blood glucose regulation is further compounded by significant inter-patient variability. However, most model-based control methods fail to capture the subtle differences between individuals fully, and FLC is even less capable of tailoring personalized control rules for each patient. The simplified mathematical models or logical rules underlying these algorithms are insufficient to represent the highly personalized glucose-insulin dynamics. Moreover, most existing control methods fall under hybrid closed-loop (HCL) algorithms, which still rely on external inputs outside the AP system, such as carbohydrate (CHO) intake, to mitigate rapid BGL fluctuations caused by external disturbances like unplanned meals. However, accurately estimating CHO intake presents a considerable challenge, often requiring specialized knowledge and training. This process significantly increases patients’ cognitive load [7] and is prone to errors [8].

**Fig 1:**
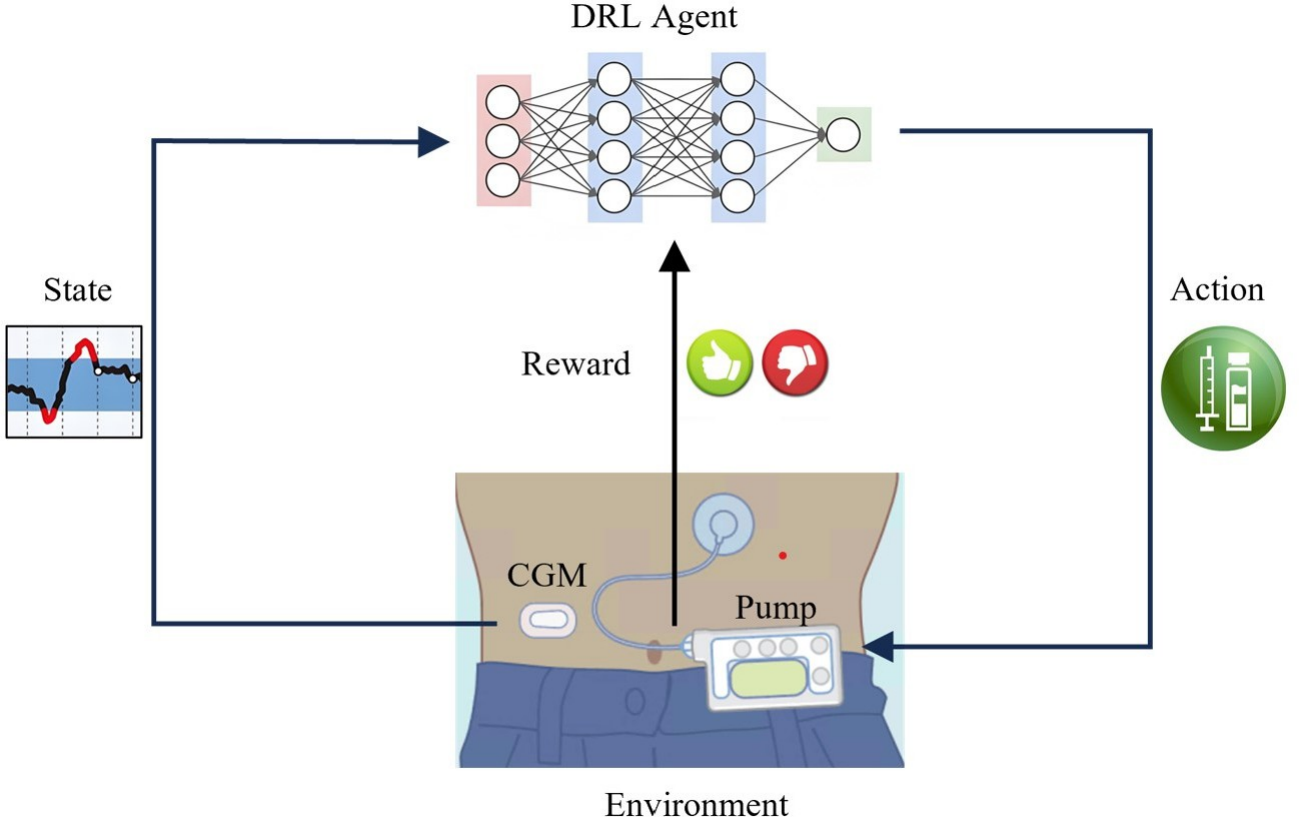
Working principle of the DRL-based AP controller.

Reinforcement Learning (RL) is emerging as a promising alternative for AP controllers [9]. In particular, Deep Reinforcement Learning (DRL), employing deep neural networks as agents, dynamically optimizes blood glucose control policies without prior knowledge of glucose metabolism models. By engaging in trial-and-error interactions with an environment comprising patients and AP system components, such as CGM devices and insulin pumps, DRL enables fully closed-loop blood glucose regulation. As illustrated in Fig 1, a DRL agent perceives the state *s* of the environment at time step *t*, represented by interstitial glucose readings measured via CGM. Based on the current policy *π*_θ_ (*a*∣*s*), the agent selects an action *a* (i.e., the insulin dosage). This action influences the environment, which transitions to a new state *s*’ according to a state transition probability *p*, and provides the agent with a reward *r*. The reward, typically derived from the next CGM reading, indicates the effectiveness of the action in regulating BGL. The agent uses this feedback to update its policy parameters from *θ* to *θ*’, perceives the new state *s*’, and repeats the process in subsequent time steps. Through iterative execution, the glucose regulation strategy is continuously refined, with insulin dosage decisions increasingly aligning with those that yield higher rewards, thereby maintaining the patient’s BGL within the normal range. The DRL agent is often implemented as a deep neural network, which excels at capturing complex patterns and representations in input data. Therefore, DRL is particularly well-suited for addressing the challenges posed by complex glucose dynamics, sensor and insulin action delays [10], uncertainties caused by daily events, and personalized requirements [11].

However, applying DRL in a clinical setting presents several key challenges, with the safety exploration dilemma at the forefront [12]. In DRL, agents continuously optimize their strategies by exploring the action space and selecting the optimal action to maximize the cumulative reward. While this ’trial-and-error’ approach works well in gaming scenarios, in safety-critical environments such as healthcare and autonomous driving, actions that could lead to irreparable harm cannot be taken, and clinical applications inevitably require safe action selection. Furthermore, the implementation of DRL-based control methods involves various system design considerations, including the selection of appropriate algorithms and their corresponding deep neural network architectures, the rational definition of the state space, the action space, and the reward function, as well as the fine-tuning of hyperparameters [13]. Different design options can significantly impact the performance of RL-based controllers and may even determine their overall success or failure. For instance, OpenAI’s default DRL algorithm, Proximal Policy Optimization (PPO), is renowned for its high performance and policy stability. However, the original PPO algorithm struggled to converge in our initial experiments without more implementation details and targeted techniques. Several implementations, such as OpenAI Baselines and OpenAI Spinning Up, recommend numerous ’tricks’ to enhance the performance of the PPO algorithm. Huang et al. compiled a list of 37 implementation details to improve algorithm efficiency [14]. This study evaluated the impact of these recommended techniques on blood glucose control performance through preliminary experiments and selected ten key tricks to enhance training stability.

Our ultimate objective is to overcome significant technical challenges to progress the clinical implementation of DRL-based AP systems. This paper marks a notable initial advancement towards this objective by developing a safe-enhanced, fully closed-loop AP system using an enhanced PPO algorithm. The key contributions can be summarized as follows:

- Ten implementation tricks are introduced to enhance the original PPO algorithm, improving its training efficiency and stability for the blood glucose control problem.
- The algorithm operates without external inputs such as meal notifications. Insulin dosages are solely determined based on recent glucose monitoring values, achieving fully closed-loop AP control.
- A dual safety mechanism with proactive and reactive measures is proposed, effectively mitigating safety risks by integrating safety considerations into the reward function and an external safety module.

The structure of the paper is as follows: Related work section provides an overview of recent advancements in related works. The proposed controller section delves into the specially designed and enhanced PPO algorithm, along with the incorporation of dual safety measures to mitigate emergencies. Performance evaluation section presents the validation of our proposed algorithm’s performance in a simulated environment. Discussion section offers a comprehensive discussion of the resolved and outstanding technical challenges our algorithm faces in facilitating the clinical adoption of fully closed-loop AP systems. Finally, we conclude with insights into future research directions.

## Related work

Despite the adaptive nature of RL to state changes and its ability to eliminate the effects of blood glucose changes, including those caused by unannounced meals, earlier proposed RL-based methods mainly focused on Q-learning approaches, where discrete insulin actions were used, and only basal insulin was controlled [15]. These systems are HCL in nature where manual estimation of the CHO content of meals and insulin dose calculations are required. The focus on autonomous DRL-based systems for glucose regulation has gained traction in recent years, with PPO and Soft Actor-Critic (SAC) algorithms demonstrating the significant potential for achieving fully closed-loop AP systems. A SAC-based system proposed by [16] used historical glucose and insulin values as input for glucose regulation. A bio-inspired RL approach using PPO has been proposed where the algorithm’s reward functions and discount factors reflect subject-specific pharmacological characteristics and temporal homeostatic objectives [17]. They both aim to eliminate CHO counting. Based on them, a control system based on PPO was proposed that controls basal and mealtime insulin infusion and requires only dietary announcements, thus eliminating the need for carbohydrate estimation [4]. On their basis, our method achieves fully closed-loop control without the need for CHO counting or any dietary announcements.

Researchers have been striving to develop DRL-based medical decision-making models that ensure safety. One proposed solution involves the establishment of a safety threshold. For instance, Thananjeyan et al. developed two modules for predicting BGL. If the expected level falls below the safety threshold within a specified time window, insulin injections are halted, and additional oral carbohydrates are administered. Conversely, if the predicted level exceeds the threshold, an extra insulin dose is given to the patient [18]. Similarly, Mackey & Furey designed an RL agent to terminate an episode when blood glucose levels deviate from the safe range, effectively averting hazardous situations. They also introduced a glycemic risk indicator to assess the performance of their proposed method [19]. However, it is essential to note that the safety threshold may vary from patient to patient, as blood glucose levels considered dangerous for some individuals may be tolerable for others. Another approach involves making decisions within physician expertise or prior knowledge constraints. For example, A decision support system for radiation therapy of glioblastoma was developed using non-invasive magnetic resonance images as input to the RL model. The system’s outputs were selected from a set of values commonly prescribed by physicians. Ultimately, the activation and termination of the system were left to the physician’s discretion, ensuring patient safety [20]. Similarly, Peng et al. combined RL model strategies with expert decision-making to learn the best strategy by limiting the agent’s prescriptions to just more than 1% of the actions taken by physicians in 300 nearest neighbors, resulting in safer drug treatment recommendations for sepsis patients [21]. Nonetheless, relying solely on constrained RL agents may limit the exploration of policies that could potentially outperform physicians or prior knowledge. Furthermore, maintaining constant physician supervision is impractical and undermines the original objective of achieving full closed-loop control. Unlike their approach, ours is based on a result-oriented idea, adopting a dual safety mechanism consisting of active and reactive measures to minimize safety risks through ex-ante incentives and ex-post adjustment.

## The proposed controller

To achieve our objective, we strive to develop a controller that emulates the regulatory mechanisms of the actual pancreas. Given the hypothesis that external factors like diet and physical activity will eventually manifest in BGL fluctuations, insulin dosage can be automatically adjusted solely based on BGL monitoring. Furthermore, rather than distinguishing between basal and bolus insulin doses, a continuously infused dose can be administered at variable rates. The code for the proposed controller has been shared on the GitHub repository: https://github.com/YanfengZhao-UKM/PPO-AP-Controller.

### A. The enhanced PPO algorithm

PPO is an advanced policy gradient algorithm that solves the problem of sensitivity to the learning rate by modifying the objective function, which restricts the magnitude of policy updates within a reasonable range. Particularly, as an optimized version, PPO-CLIP introduces a simple clipping mechanism to constrain the magnitude of policy updates, as in

$$L^{Clip}\;(\theta )=E[min\;(r(\theta )*A,clip(r(\theta ),1-\varepsilon ,1+\varepsilon )*A)]$$ (1)

where *A* is the advantage function; $r(\theta )=\frac{\pi_{\theta }(a|S)}{\pi_{\theta^{'}}(a|S)}$ represents the ratio of the probabilities of taking action *a* given state *s* under the current and previous policies. *r* > 1 indicates that the new policy increases the probability of taking action *a* given state *s*, while *r* < 1 indicates that such a tendency decreases; the function *clip*(*) is the clipping mechanism to restrict excessive updates.

Despite the remarkable performance and stability of the PPO algorithm, we encountered challenges when applying it to highly complex AP control problems during our initial experiments. This improved it by incorporating ten tricks inspired by prior research [14, 22] and extensive experimental exploration.

#### 1. Beta probability density function

**Fig 2:**
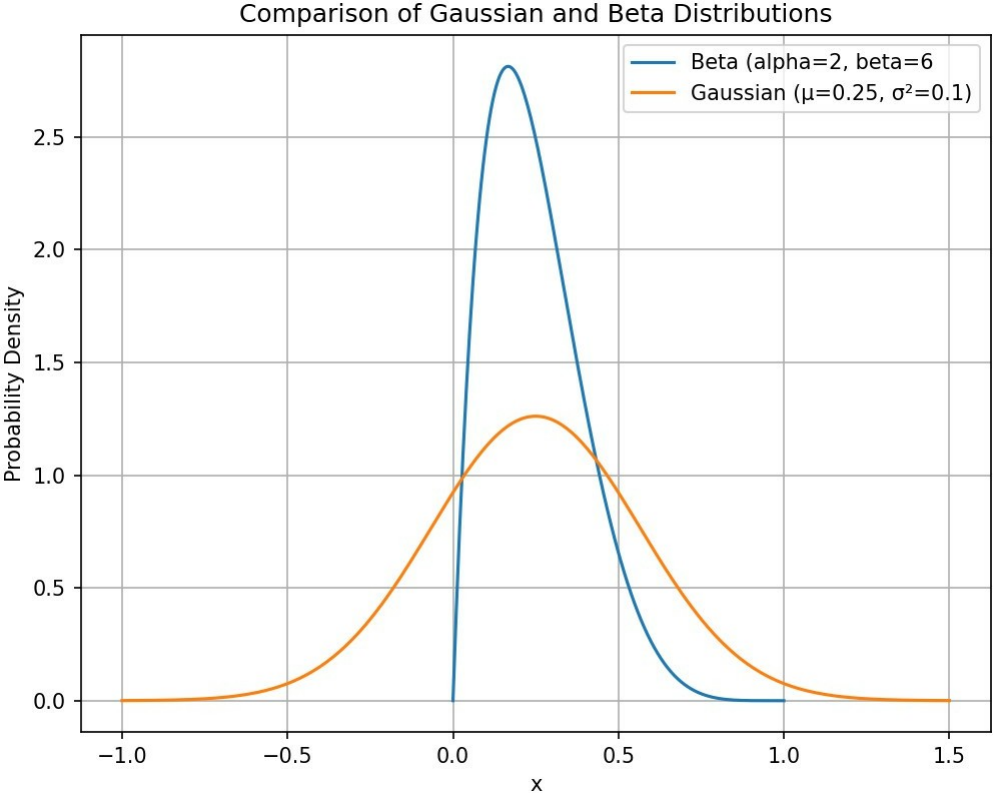
Comparison of Beta distribution and Gaussian distribution.

The PPO algorithm defaults to using the Gaussian distribution to produce actions. However, due to its unbounded nature, a Clamp function is often necessary to confine the sampled actions within the specified action space range. Yet, this clamp operation indiscriminately adjusts all actions outside the range to the bounded values of the action space, inadvertently elevating the likelihood of selecting the maximum and minimum values. To mitigate this issue, Chou et al. [23] propose employing a bounded Beta distribution as an alternative to the Gaussian distribution, thereby confining the selected actions to the [0,1] interval (Fig 2). In the AP control environment, where insulin doses must be non-negative and excessive instantaneous doses pose risks, a Beta distribution capable of constraining the sampled actions to the desired range offers clear advantages.

#### 2. Advantage function normalization

Tucker et al. [24] believed that normalizing the advantage function can enhance the performance of the policy gradient algorithm. After experimental comparisons, we implemented their idea using a batch_adv_norm method, which begins with deriving all the advantages within a batch using generalized advantage estimation (GAE), followed by computing their mean and variance and then normalizing them using the traditional method of subtracting the mean and dividing by the variance.

#### 3. Treatment failure indicator

Under typical circumstances, despite fluctuations or significant deviations in BGL, the AP system generally manages to sustain treatment until the end of an episode. The termination of treatment is typically an artificially imposed truncation of the ongoing episode initiated for iterative policy refinement. Even though it has reached the ’done’ state, a subsequent state still exists. However, in cases where AP control is ineffective, and BGL persistently deteriorates, potentially resulting in emergencies or even fatalities, reaching the ‘done’ state signifies treatment failure. In such instances, the patient does not transition into the subsequent state. To distinguish between these completely distinct ’done’ states, we introduce a failure identifier to separate the GAE calculation when updating the policy.


$$GAE^{\prime }=\left\{\begin{array}{cc} r+\gamma *v(s\prime )-v(s)+\gamma *\lambda *GAE & if\;done=0\\ r-v(s) & if\;done=1\;and\;dw=1\\ r+\gamma v(s\prime )-v(s) & if\;done=1\;and\;dw=0 \end{array}\right.$$ (2)

Here, GAE and GAE’ represent the current and updated Generalized Advantage Estimation values, respectively. r denotes the reward value of the current timestep, while v(s) and v(s’) represent the value estimates of the current state and the next state, respectively. ’dw = 1’ indicates treatment failure, whereas ’dw = 0’ indicates that the maximum timestep of an episode has been reached.

#### 4. State normalization

As mentioned, the proposed algorithm updates policies by sampling one batch of data at a time. However, different batches of data may have varying maximum and minimum values. Given that we utilize one-hour BGL monitoring records as the state of the PPO controller, these differences can be very considerable. Consequently, traditional normalization methods may lead to the same BGL in different batches of data being normalized to distinct state values. To address this issue, we adopted a simple normalization method tailored to the trait of BGL data falling within the range [0, 600]. Specifically, we divided each BGL value by its maximum value. This approach ensures the consistency and validity of the normalization results across different batches.

#### 5. Policy entropy

Entropy is a measure that quantifies the uncertainty associated with a random variable. In the context of DRL, the entropy of a policy can be expressed as follows:

$$H\left(\pi \left(\cdot |s_{t}\right)\right)=E_{a_{t}\sim \pi }\left[-\log\;\left(\pi \left(a_{t}|s_{t}\right)\right]\right.$$ (3)

#### 6. Learning rate decay

A large learning rate during the DRL model training process can result in unstable training, while a small one may reduce training efficiency. We implement a linear decay of the learning rate to address this dilemma. This approach entails gradually decreasing the learning rate from the initial value of 2e-5 as the number of training steps progresses, eventually reaching 0. This linear decay promotes smoother transitions in the later stages of training, to some extent enhancing training effectiveness.

Additionally, we utilized Reward Normalization, Gradient Clipping, Orthogonal Initialization, and Adam Optimizer Epsilon Parameter as our algorithmic enhancement tricks, which we will not elaborate on here due to space limitations. We have configured these enhancements as command-line parameters to provide flexibility when setting them at runtime. This facilitates comparing the proposed controller’s performance enhancements after incorporating these tricks. Several other tricks proposed in the literature were initially considered but discarded after preliminary experiments demonstrated limited effectiveness.

### B. The dual safety mechanism

Our safety design principle aims to maintain the final infusion dose within a safe range while preserving the DRL controller’s ability to explore optimal strategies without constraints. To achieve this, we have devised a dual safety mechanism termed ’proactive guidance + reactive correction,’ grounded in the outcomes of glucose regulation. Before action selection, a specifically crafted reward function directs the controller towards actions conducive to glucose stability. Following action selection, an independent safety adjustment module evaluates the current BGL and its trend to make dosage decisions.

#### 1. Reward function design

The design of the reward function is crucial for the performance of the DRL model, especially in complex environments such as diabetes management. A poorly designed reward function can struggle to incentivize satisfactory policies. We conducted experiments comparing various reward schemes proposed in previous research and made specific improvements based on our design goals. As a result, the final reward function differs from any previous studies.

Firstly, our reward is based on the Clarke glucose risk index (RI) [25] rather than simply segmenting BGL ranges. This choice stems from our desire not only to maintain BGL within the normal range of 70–180 mg/dL but also to guide them towards an ideal glucose value (112.517 mg/dL)—Clarke’s zero-risk point for glycemic control—while keeping them stable. However, defining the reward function simply as the negative value of RI presents a challenge. Negative reward values would encourage the DRL controller to end each episode early to maximize cumulative rewards, potentially prematurely putting the patient in an emergency and terminating treatment. Some propose incorporating a sizeable negative value into the reward function to penalize treatment failures. However, empirical evidence shows that a broad range of reward values is not ideal. Therefore, we set the instantaneous reward within the (0,1) range.


$$r={\left(1-\frac{RI}{180}\right)}^{2}$$ (4)

Where RI refers to the glucose risk index calculated according to the Clarke method, the parameter 1/180 aims to normalize the RI to the (0,1) range. Subsequently, the reward value is set to be negatively correlated with the RI, and the final square operation aims to amplify the impact of BGL deviations to enhance the effectiveness of the reward.

So far, we have achieved the goal that the greater the deviation from the ideal BGL, the higher the RI, resulting in a smaller immediate reward. This itself helps prevent BGL from being excessively high or low. Building upon this, to guide the controller towards selecting safer dosages, we have intensified the penalties for BGL values outside the [70,180] range, particularly in the most dangerous cases of severe hypoglycemia (S_hypo, BGL<50 mg/dL). The final reward function is as follows:

$$R=\left\{\begin{array}{cc} r & if\;70\leq BGL\leq 180\\ r\times 0.5 & if\;50\leq BGL<70\;or\;BGL>180\\ 0 & if\;BGL<50 \end{array}\right.$$ (5)

Here, distinct rewards are set for BGL within the range, outside the range, and in S_hypo conditions. Rewards for hyperglycemic and hypoglycemic are halved, while the reward for S_hypo is set to 0. This aims to guide the DRL controller in reducing the probability of selecting actions that may lead to these states, thereby minimizing the occurrence of high-risk situations.

#### 2. Safety adjustment module

Although safety considerations are embedded in the reward function design, the fundamental nature of the DRL controller, based on ’trial and error’ through random sampling of actions, does not guarantee that the insulin doses selected each time are suitable for the patient’s current state. Additionally, a fully closed-loop controller cannot be aware of external disturbances such as carbohydrate intake and thus cannot immediately respond to sudden changes in BGL caused by them. To address these challenges, we have implemented a safety adjustment module. This module calculates a coefficient, Insulin_coef, based on the current time of day and the patient’s BGL monitoring records. This coefficient adjusts insulin doses promptly to adapt to the patient’s current BGL status and potential upcoming BGL anomalies.

First, we need to address the issue of nocturnal hypoglycemia, which is a common concern for diabetes patients. However, since the time variable is not included in the state input to the DRL controller, it does not inherently learn policies to deal with this problem. Our solution is to monitor the current time in the safety adjustment module. We set the insulin adjustment coefficient to 0.8 after 8 p.m. each night and 0.6 before 3 a.m. to reduce nocturnal insulin infusion and alleviate this issue. The determination of these coefficients follows the recommendations in the medical guidelines [26].

Next, based on one-hour BGL monitoring records, we analyze the deviation of the current BGL (the ratio of the last measurement value to the ideal value) and its trend (the ratio of the previous two measurement values). We then calculate the insulin adjustment coefficient according to the following formula:

$$coef=\frac{obs[0]}{112.517}\times {\left(\frac{obs[0]}{obs[1]}\right)}^{2}$$ (6)

Where *obs*[0] and *obs*[1] represent the last and second-to-last CGM readings, respectively, and 112.517 is the ideal BGL value. If the current BGL deviation, $\frac{obs[0]}{112.517},$is greater than 1, it indicates that the BGL is high, so the insulin dose should be increased accordingly. Otherwise, decrease the insulin dose as BGL is low. Similarly, if $\frac{obs[0]}{obs[1]}$ is greater than 1, it means that the BGL is rising, and the insulin dose should be increased; otherwise, decrease the insulin dose as BGL is falling. Squaring the rate of change of BGL amplifies its impact on the insulin dose.

Finally, most cases of hypoglycemia can only be improved through CHO intake, and the closed-loop controller must make every effort to prevent the patient from experiencing hypoglycemic states. Therefore, when the current BGL is less than 80, the insulin adjustment coefficient is set to 0, halting insulin infusion to reduce the risk of hypoglycemia.

### Performance evaluation

The proposed controller’s performance was evaluated using Simglucose 2018 [27], and the results were compared with those of a hybrid closed-loop control algorithm and the state-of-the-art DRL-based fully closed-loop control algorithm.

#### A. Experimental setup

For the glucose regulation performance evaluation, 30 virtual patients (10 adults, 10 adolescents, and 10 children) were used as test subjects, while only the virtual patient ’adolescent#001’ for exploratory experiments. The ’Dexcom’ device was selected as the CGM system in the simulation environment, and its measurement errors were modeled using Gaussian noise. The ’Insulet’ was chosen as the insulin pump, with its default dosing range set to [0, 30] units. The simulator operates with a default time step of 3 minutes, with each episode consisting of 480 steps, representing one day. Evaluation metrics include cumulative reward, percentages of time in range, hyperglycemia, and hypoglycemia. Table 1 lists some of the hyperparameters set in the experiments, provided as reference values for reproducing the study. The hyperparameter settings adhere to general recommendations for algorithm implementations and are similar to the values reported in [4, 17]. The experiments demonstrate that adjusting these values within a reasonable range does not significantly impact outcomes.

**Table 1:** Hyperparameters in the experiments.

Hyperparameter	Value
**Max episode steps**	480
**Batch size**	128
**Minibatch size**	16
**Initial learning rate**	2*10^−5^
**Discount factor (gamma)**	0.999
**GAE parameter (lambda)**	0.95
**PPO clip range (epsilon)**	0.1
**No. of policy epochs**	20
**Entropy Coefficient**	0.02

According to the Guidelines for the Diagnosis and Treatment of Type 1 Diabetes Mellitus in China (2021 edition), we calculated daily CHO intake based on the patient’s age and weight, setting daily CHO intake for adults between 290 and 400 grams, for adolescents between 220 and 310 grams, and for children between 150 and 250 grams. The distribution ratios for adults’ breakfast, lunch, dinner, and bedtime snacks were 5:6:5:4, while minors’ main meals and snacks were distributed according to a 4:2:5:2:4:3 ratio. To closely mimic the clinical setting, we introduced a random variation of ±20% in meal size and ±30 minutes in meal timing for each meal.

#### B. Effect of algorithm enhancement

**Fig 3:**
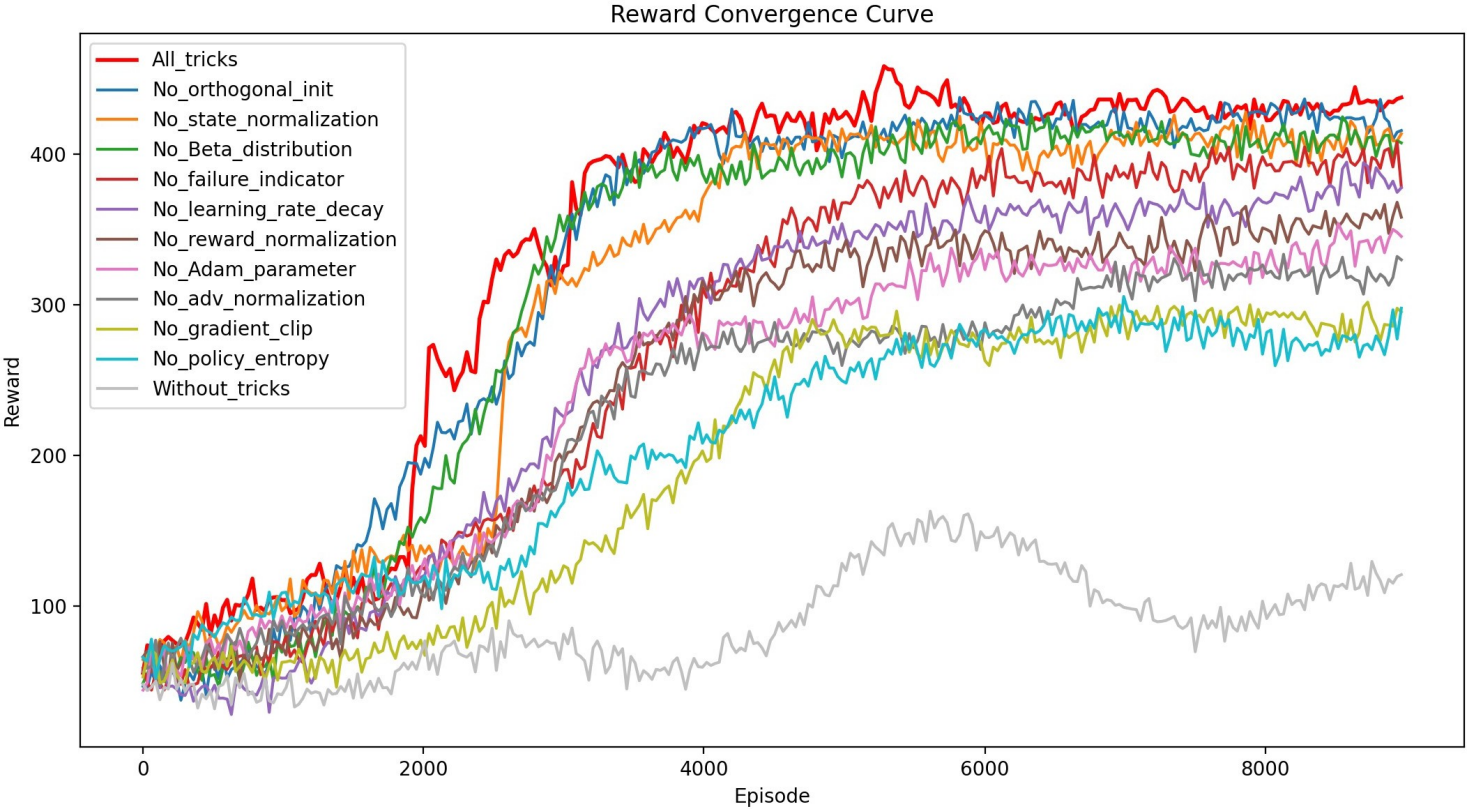
Comparison of convergence curves after using different tricks.

Ablation experiments were conducted to assess the impact of different combinations of optimization tricks on training performance. Each combination was used to train the virtual patient ’adolescent1’ over 3.6 million time steps, with cumulative rewards recorded for each episode. As shown in Fig 3, the PPO algorithm, without any tricks, failed to converge. In contrast, using all ten tricks significantly improved both the performance of blood glucose control and the training stability, with cumulative rewards exceeding 400 per episode after approximately 4,000 episodes and maintaining stability after that. Among the optimization tricks, policy entropy and gradient clipping had the most significant impact on training efficiency, while orthogonal initialization, state normalization, and the Beta distribution showed minimal effects on model convergence. Due to treatment failures, the number of training episodes varied across the combinations, so the first 9,000 episodes were compared. Given the extended training time—exceeding 21 hours for each combination—the ablation experiments were restricted to a single virtual patient.

#### C. Glucose regulation results

**Fig 4:**
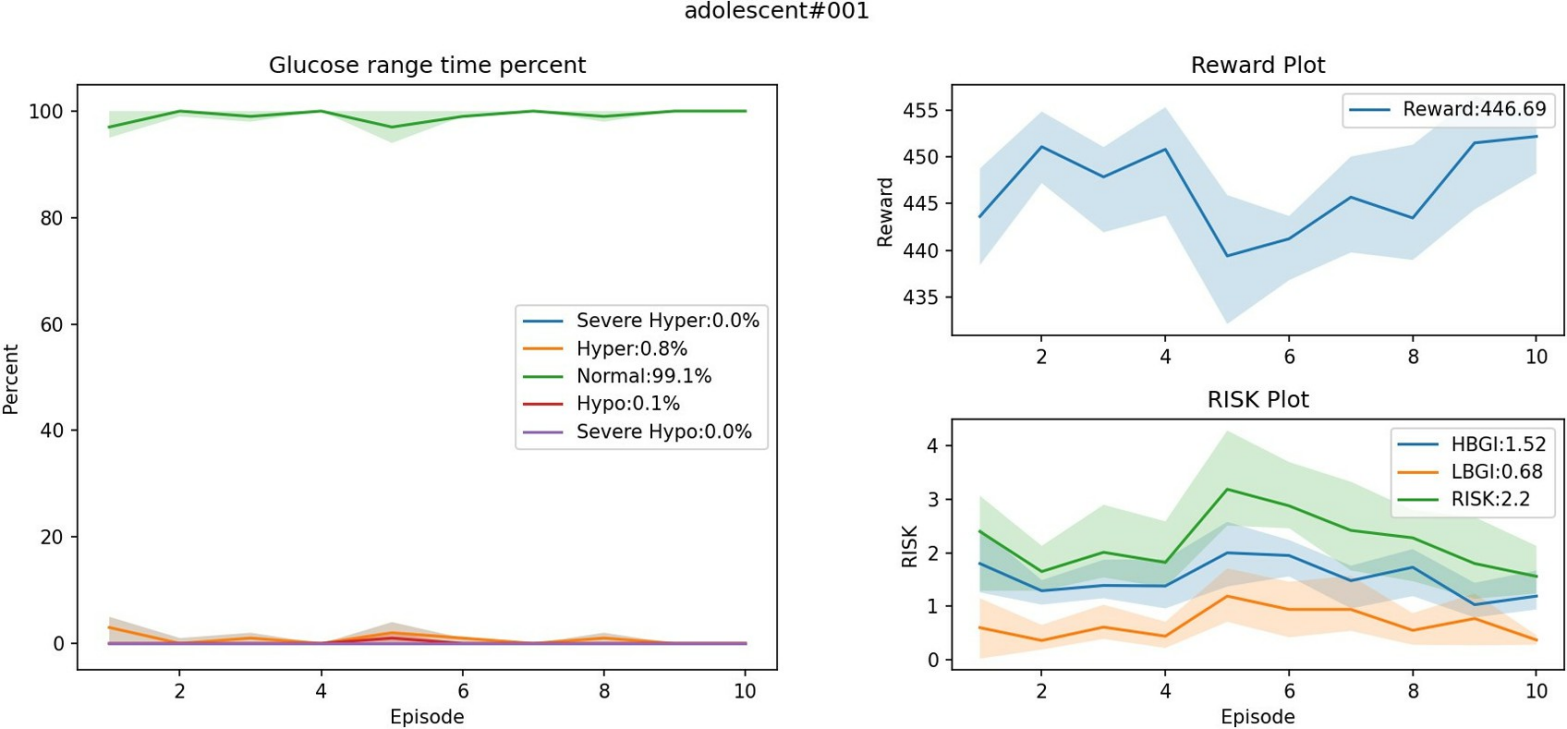
Glucose regulation results for ’adolescent#001’.

Separate control policies were trained for each virtual patient using the improved algorithm. Subsequently, 10 days of monitoring data were recorded, capturing key metrics such as the percentage of time in range (TIR, 70–180 mg/dL), time above range (TAR, >180 mg/dL), time below range (TBR, <70 mg/dL), time spent in severe hyperglycemia (S_hyper, >300 mg/dL) and S_hypo, as well as daily cumulative reward and RI, to evaluate the effectiveness of the control policies. Fig 4 demonstrates the glucose regulation results of the ’adolescent#001’ control policy.

The performance evaluation metrics for all virtual patients are presented in Table 2. The experimental results indicate that the proposed control algorithm met expectations for all virtual patients. Specifically, the percentage of TIR exceeded 76%, with a median of 87.45(79.90–91.45). The incidences of hyperglycemia and hypoglycemia were 11.75(6.30–17.80) and 0.75(0.40–1.05) respectively, and the RI consistently remained at a low level of 5.77(4.49–6.84). Notably, S_hyper and S_hypo, which pose emergency risks to the patient, were virtually eliminated, which means that there were no treatment failures during the assessments, effectively achieving the goal of safety assurance.

**Table 2:** The performance evaluation metrics for all virtual patients.

Patient	TIR (%)	TAR (%)	TBR (%)	RI
**Median with IQR**	**87.45 (79.90–91.45)**	**11.75 (6.30–17.80)**	**0.75 (0.40–1.05)**	**5.77 (4.49–6.84)**
**adolescent#001**	99.1	0.8	0.1	2.2
**adolescent#002**	77.3	21.8	0.9	6.85
**adolescent#003**	90.6	8.7	0.5	4.91
**adolescent#004**	87.0	11.9	1.4	5.82
**adolescent#005**	79.4	19.9	0.7	6.33
**adolescent#006**	88.9	10.5	0.6	4.84
**adolescent#007**	83.1	15.9	0.9	5.89
**adolescent#008**	89.5	8.4	2.0	6.04
**adolescent#009**	94.3	4.6	1.0	4.23
**adolescent#010**	84.5	14.7	0.7	6.07
**adult#001**	95.3	4.0	0.7	4.19
**adult#002**	97.8	1.9	0.2	2.97
**adult#003**	92.2	7.4	0.3	4.35
**adult#004**	77.2	21.4	1.5	7.55
**adult#005**	91.5	8.1	0.3	4.01
**adult#006**	79.5	19.3	1.1	6.51
**adult#007**	79.5	19.8	0.8	7.04
**adult#008**	97.5	2.6	0.1	2.88
**adult#009**	88.5	11.1	0.2	4.49
**adult#010**	88.2	11.3	0.4	4.97
**child#001**	82.0	17.3	0.7	6.57
**child#002**	91.2	7.7	1.0	5.01
**child#003**	78.5	20.4	0.9	6.94
**child#004**	76.7	21.4	1.7	7.64
**child#005**	87.9	11.6	0.7	5.15
**child#006**	77.6	21.1	1.3	6.84
**child#007**	90.3	9.6	0.1	4.33
**child#008**	82.3	16.0	1.8	6.79
**child#009**	84.6	14.5	0.8	5.71
**child#010**	78.5	20.1	1.5	7.0

#### D. Comparison with the baseline

A relatively mature HCL-based PID controller and a fully closed-loop DRL-based controller were compared to evaluate their performance in regulating BGL. The PID algorithm was used in the first commercially approved HCL system, the Medtronic 670G insulin pump, and its safety and efficacy were clinically validated. We implemented a PID controller and tested it in the Simglucose environment. Similar to the proposed algorithm, we monitored the glucose regulation results of 30 virtual patients, recording metrics such as TIR, TAR, TBR, and IR, with a particular focus on the percentages of S_hyper and S_hypo, and the incidence of treatment failure. The main difference is that the PID controller was informed of CHO intake during glucose regulation.

For the fully closed-loop DRL-based controller, we compared our results with those reported in existing studies, selecting benchmarks based on the following criteria: 1) fully closed-loop blood glucose regulation without any meal announcements; 2) testing conducted on all 30 virtual patients; 3) evaluation metrics included at least TIR, TAR and TBR, preferably also IR and treatment failure incidence; 4) experimental settings closely matching ours. Based on these criteria, the results reported in [16] were chosen as the baseline, where fully closed-loop glucose control was achieved using the SAC algorithm. Several other experimental results from the literature will be discussed in the next section.

**Fig 5:**
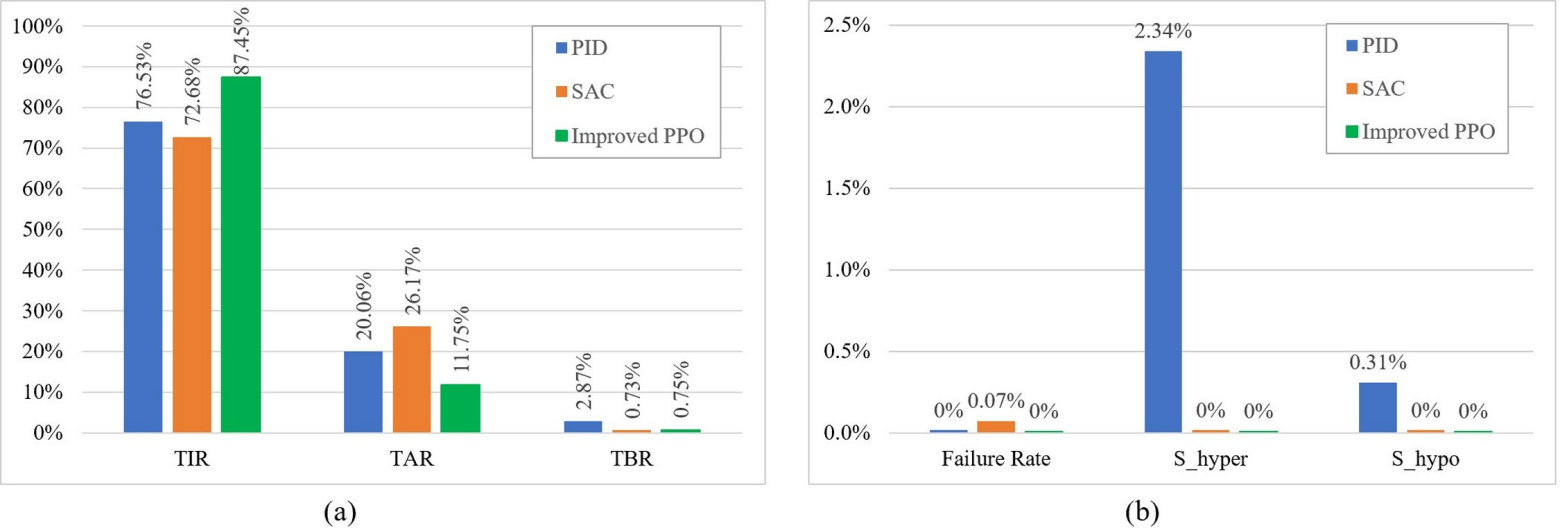
Performance comparison of the proposed algorithm with the baseline. (a) The proposed algorithm achieved superior TIR and TAR compared to both baselines. Although the TBR was slightly higher than SAC, it was significantly lower than that of PID. (b) The proposed algorithm eliminated emergencies and treatment failures, a goal neither baseline achieved.

Fig 5 compares the glucose regulation performance of the proposed controller with the two baselines. In terms of effectiveness, the improved PPO controller achieved TIR median improvements of 10.92% and 14.77% compared to the PID controller with meal announcements and the fully closed-loop SAC algorithm, which had medians of 76.53% (70.3–81.2) and 72.68% (67.7–76.2), respectively. The TAR median decreased by nearly half or more relative to both baselines (20.06% [15.8–29.0] for PID and 26.17% [23.1–30.6] for SAC). The proposed controller achieved a slightly higher TBR median than the SAC algorithm (0.73% [0.0–1.8]) but showed significant improvement over the PID controller’s hypoglycemia rate (2.87% [1.4–5.6]). Additionally, the proposed controller demonstrated superior IR performance, outperforming both the PID (7.76 [4.7–11.6]) and SAC (6.50 [4.8–9.3]) algorithms. In terms of safety, the proposed controller completely avoided S_hyper and S_hypo, with no treatment failures observed during the 10-day evaluation of 30 virtual patients. In contrast, the PID algorithm, although free of treatment failures, exhibited 2.34% S_hyper and 0.31% S_hypo. The study in [16] did not report the incidence of S_hyper and S_hypo but did show a 0.07% treatment failure rate.

## Discussion

Our ultimate goal is to develop a fully closed-loop AP system and to overcome the main technical obstacles to its clinical application. This paper marks an important first step towards this goal by designing a safety-enhanced, fully closed-loop DRL-based controller, achieving two fundamental objectives: 1) We have realized fully closed-loop glucose control, managing insulin infusion and automatic glucose regulation for T1DM patients based solely on the past hour’s BGL monitoring data without requiring any additional information. This approach eliminates external errors, particularly those arising from estimating CHO intake. 2) We have introduced a dual safety design of ’proactive guidance + reactive correction,’ which controls insulin infusion doses based on glucose regulation results to keep them within a safe range, minimizing the risk of emergencies.

**Fig 6:**
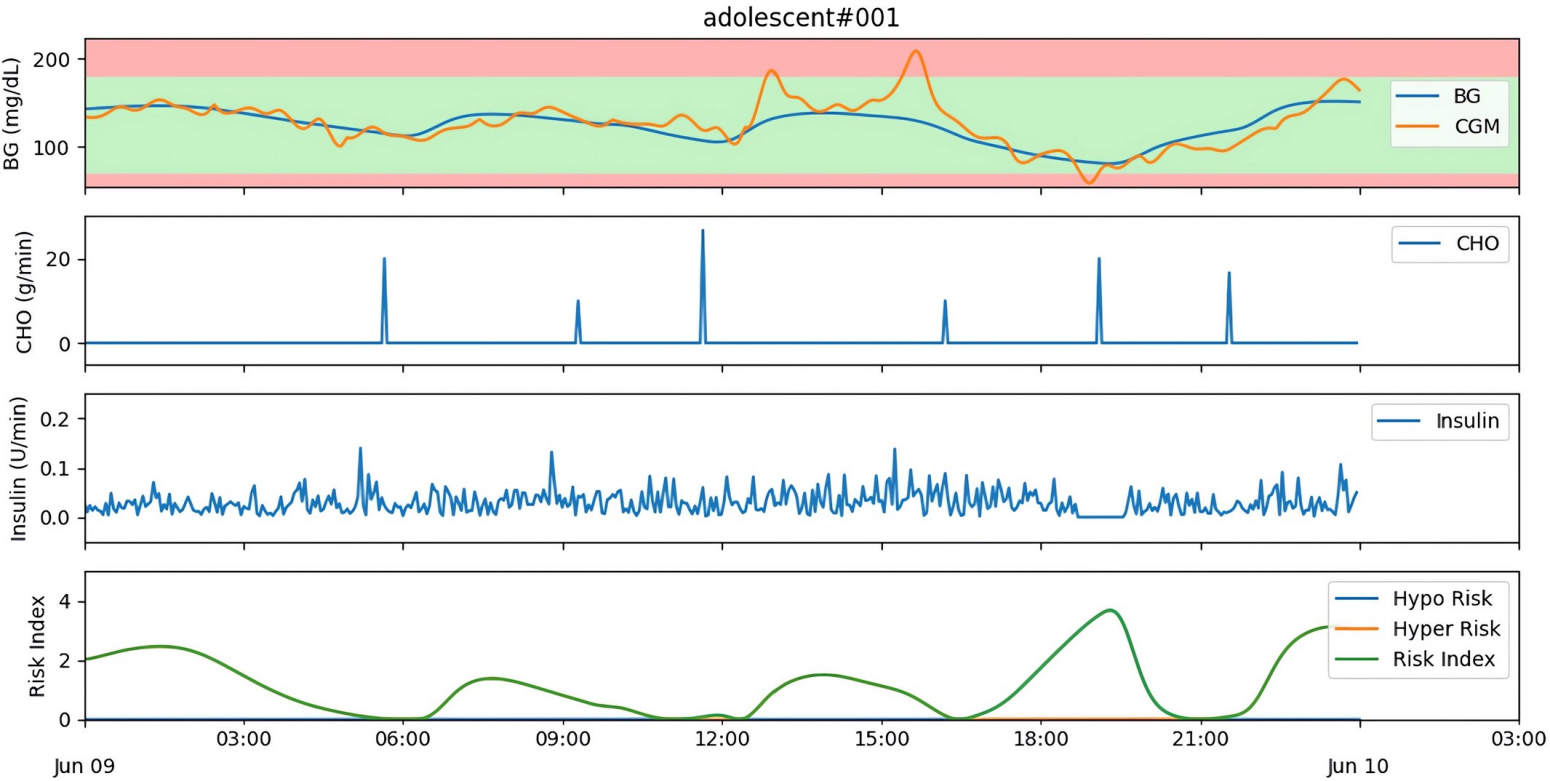
Treatment records for ’adolescent#001’ in one day.

Through animation, we tracked ’adolescent#001’ for a day, monitoring BGL, CGM readings, CHO intake, insulin dosage, and RI values (Fig 6). The analysis showed that the insulin dose increased accordingly when CGM readings were too high or increased rapidly (1–4 pm). The insulin dosage remained low at night, and insulin infusion was halted when CGM readings were too low (around 7 pm). However, the insulin dosage did not always directly correspond to CGM readings, nor was there a clear direct relationship with CHO intake. Specifically, the insulin peak did not occur within a few minutes after each meal. Instead, the peak occurred approximately half an hour before the meal for breakfast and morning snacks, likely due to the fixed meal times set at around 6:00 AM and 9:00 AM. The control model may have learned this pattern and made anticipatory adjustments. However, insulin infusion became more concentrated for lunch and afternoon snacks, likely influenced by inevitable errors in CGM measurements. In the dinner phase, insulin even paused, which was clearly a result of previous excessive infusions leading to low CGM readings. This indicates that the trained model effectively captures the patient’s glucose dynamics without solely relying on the safety module. This further demonstrates the effectiveness and safety of the proposed controller, even in the presence of measurement errors and meal disturbances.

Multiple evaluation metrics were employed to measure the glucose regulation performance of the proposed controller. Simulation experiments conducted in Simglucose revealed that the proposed controller achieved satisfactory results in both effectiveness and safety. Without meal announcements, it attained a higher TIR of 87.45(79.9–91.5) compared to the baselines PID controller (76.53 [70.3–81.2]) and SAC controller (72.68% [67.7–76.2]), and about half the TAR at 11.75(6.3–17.8). The TBR median was kept within 1%, peaking at only 2.0%. Notably, no treatment failures occurred in the 10-day test involving 30 patients, and neither S_hyper nor S_hypo states were observed. Medical experts confirmed these results as highly encouraging, although around 30% of the patients showed TAR values close to or exceeding 20%. We attribute this success to the technical improvements made to the PPO algorithm and the unique safety design of our controller. Specifically, choosing a Beta distribution instead of a Gaussian for the probability density function significantly reduced uncertainty during the action sampling process, enabling the DRL controller to determine the optimal dose for specific states quickly. The maximum insulin dose determined by the controller was just 0.52 units during testing. This moderate dose helps prevent drastic fluctuations in BGL, indicating that the enhanced functionality not only significantly accelerated the convergence and stability of training but also improved blood glucose regulation performance. The well-designed reward function not only aimed to maintain BGL within the normal range but also to progressively approach the ideal value, minimizing the risk of hyperglycemia and hypoglycemia. After the algorithm makes a choice, the safety adjustment module further refines the dose based on the current BGL and its trend, ensuring the high performance and safety of the DRL controller.

Some evaluation metrics reported in the literature surpass our results. For instance, Lee et al. [17] reported a TIR of 89.56%, and the ARLPE framework proposed in another study [28] achieved TIRs of 98.63% and 97.93% in adults and adolescents, respectively. However, it is important to note that Lee et al. tested only 10 virtual adult patients, and the performance of the ARLPE framework has not been validated in pediatric patients. It is well known that minors, especially children, are much more sensitive to external insulin interference, which likely results in less favorable test outcomes compared to adults. Moreover, our simulations revealed that lower CHO intake often yields better experimental results, but doctors do not recommend overly low meal quantities as they may lead to dangerous hypoglycemia and malnutrition. In the study by Lee et al., the daily CHO intake for adults was fixed at 180 grams, which, according to the Guidelines, is insufficient for 4-8-year-old children and certainly does not meet the nutritional needs of adults. The meal quantity data was missing in [28], and CHO intake estimation and notification were still required. In contrast, we strictly followed the Guidelines to customize meal quantities for virtual patients and to set different meal frequencies for adults and minors. Medical experts consider this assignment more reasonable than those reported in previous studies.

However, our initial solution is not without flaws and has a long way to go before clinical application. One significant obstacle that needs to be addressed is the interpretability of the DRL model. Although the effectiveness and safety of the proposed controller were validated through simulation experiments, there is no theoretical proof or necessary rational explanation for why it can make correct decisions or whether the decisions are optimal. Our DRL controller essentially functions as a black box, using deep neural networks to map BGL monitoring values directly to insulin doses without providing a causal relationship between these state features and the selected actions. This inherent defect makes its decisions challenging to understand, thus hindering patient trust and acceptance. Physicians are also unlikely to assume significant risks by implementing treatments that lack transparency, correctness, safety, and robustness [29]. Despite the growing emphasis on the interpretability of machine learning algorithms in the medical field [30, 31], existing feature importance-based explanation methods are more suited to predictive models and are challenging to apply to control or decision models. Consequently, there are few solutions provided in the literature. Lim et al. [32] leverage two attention modules to emphasize the significance of variables and time series, thereby shedding light on the intentions and behavior of the model from an interpretability standpoint. Lee et al. [17] used the Layer-wise Relevance Propagation technique to analyze the correlation between insulin infusion rates and BGL, glucose change rates, and estimated onboard insulin. These model explanation techniques are becoming promising methods for providing healthcare professionals with insights into the relationship between DRL model inputs and outputs [33]. However, these methods have limited ability to enhance the decision transparency of DRL-based control models [34]. Therefore, finding a suitable explanation technique to identify potential biases in the model and improve the interpretability and credibility of machine decisions is a priority for future research.

The limitations of the proposed controller also include its low training efficiency. Despite algorithmic improvements that facilitate model convergence, training an optimal policy for an individual patient still requires over 4000 episodes. This translates to potentially more than a decade of real-world time to discover a satisfactory control policy, during which frequent treatment failures would be intolerable. Recent studies have suggested using offline RL to address this issue [35, 36]. However, it is essential to note that while offline RL alleviates the ethical concerns of real-world clinical trials, the advent of simulators has already mitigated these worries to some extent. A more pressing issue is how to transfer policies trained on datasets to actual patients, given the variability in glucose dynamics and insulin responses among individuals. Trained policies often prove effective only for specific patients, necessitating a unique policy for each new patient to meet these personalized requirements. Like us, [37] has also recognized the issue of policy transferability. In future research, we plan to incorporate transfer learning to enable policy transfer between highly correlated patients. The meta RL framework proposed by Yu et al. [28], which combines general policies with personalized fine-tuning, could serve as a valuable inspiration for our solution.

Table 3 summarizes the technical challenges that have been resolved and those that remain unresolved in the clinical application of the fully closed-loop AP controller in this study. This highlights the proposed controller’s strengths and weaknesses and directions for future research.

**Table 3:** Technical challenges resolved and unresolved in this study.

No.	Technical Challenge	Resolved	Solution/Optional solutions
**1**	Fully closed-loop	Y	DRL-based controller
**2**	Safety	Y	A dual safety mechanism
**3**	Training stability	Y	Enhanced PPO with ten tricks
**4**	Interpretability	N	Explainable artificial intelligence technology
**5**	Transferability	N	Meta-learning or transfer learning

## Conclusion

This study proposes a DRL-based AP controller that achieves adaptive glucose regulation using only the recent CGM recordings without relying on external information such as meal notifications. Its advantages are mainly attributed to two aspects: 1) the introduction of ten tricks to improve the state-of-the-art PPO algorithm, making it more suitable for capturing complex glucose dynamics and facilitating rapid convergence and performance enhancement during model training. 2) A well-designed reward function that actively guides the patient’s BGL to be as close as possible to the ideal value and away from emergencies, while an external safety module adjusts insulin doses based on the current BGL, implementing dual safety assurances. A ten-day test on 30 virtual patients showed that the proposed controller achieved a TIR median of 87.45(79.9–91.5), a TAR median of 11.75(6.3–17.8), and a TBR median of 0.75(0.4–1.1), with no emergency events or treatment failures. These results demonstrate superior effectiveness and safety compared to the two baselines, illustrating the potential of the proposed AP controller for further clinical application. Future studies will focus on enhancing the interpretability of the DRL controller to build confidence for clinical applications and on using transfer learning techniques to port the trained strategy to a broader range of new patients, further improving training efficiency.
